# Antipsychotics are related to psychometric conversion to psychosis in ultra‐high‐risk youth

**DOI:** 10.1111/eip.13158

**Published:** 2021-05-05

**Authors:** Antonio Preti, Andrea Raballo, Anna Meneghelli, Angelo Cocchi, Maria Meliante, Simona Barbera, Lara Malvini, Emiliano Monzani, Mauro Percudani

**Affiliations:** ^1^ Programma2000 – Center for Early Detection and Intervention in Psychosis, Department of Mental Health Niguarda Ca' Granda Hospital Milan Italy; ^2^ Department of Neuroscience University of Turin Turin Italy; ^3^ Section of Psychiatry, Clinical Psychology and Rehabilitation, Department of Medicine University of Perugia Perugia Italy; ^4^ Center for Translational, Phenomenological and Developmental Psychopathology (CTPDP) Perugia University Hospital Perugia Italy

**Keywords:** early intervention, psychosis, survival analysis, ultra‐high‐risk

## Abstract

**Background:**

The prescription of antipsychotics outside overt psychotic conditions remains controversial, especially in youth where it is relatively widespread. Furthermore, some studies seem to indicate that antipsychotic exposure in individuals at ultra‐high‐risk (UHR) for psychosis is associated with higher conversion rates. This study was set up to test whether the inter‐current prescription of antipsychotics in UHR patients was related to the psychometric threshold for a diagnosis of psychosis.

**Methods:**

The 24‐item Brief Psychiatric Rating Scale (BPRS) was used to quantify treatment response up to 2 years in 125 UHR participants. Standard psychometric criteria were used to quantify conversion to psychosis. Kaplan‐Mayer and Cox proportional hazard survival analysis were applied to determine the impact of having or not received the prescription of an antipsychotic drug.

**Results:**

Over the study period 30 (24%) subjects received the prescription of an antipsychotic. In the sample, there were 31 participants (25%) who had reached the psychometric threshold for conversion to psychosis after 2 years of treatment. UHR people who received a prescription of antipsychotics during the first 2 years of treatment were statistically more likely to reach the psychometric threshold for conversion to psychosis on the BPRS: Hazard ratio = 3.03 (95%CI: 1.49–6.16); *p* = .003.

**Conclusion:**

This finding supports the hypothesis that the prescription of antipsychotics within UHR cohorts is to be considered a red flag for higher incipient risk of conversion to psychosis.

## 1 INTRODUCTION

Whilst there is indubitable, multilevel evidence for the effectiveness of antipsychotics in the treatment of psychosis (Ceraso et al., 2020; Zhu et al., 2017), this is still not the case for subthreshold risk syndromes such as ultra‐high‐risk (UHR) for psychosis (Yung et al., 2004; Yung & McGorry, 1996). So far, two small sample size trials have shown that low‐dose antipsychotics can reduce prodromal psychopathology and delay the onset of psychosis in UHR patients (McGlashan et al., 2006; McGorry et al., 2002). However, the protective effects of low‐dose antipsychotics in UHR patients are not long lasting and fade away when controlled at 12‐month follow‐up (McGlashan et al., 2006; McGorry et al., 2013). Overall, the prescription of antipsychotics in youth remains controversial and critically depends on cost/benefit ratio. Indeed, antipsychotics are often prescribed in youth for nonpsychotic disorders and for conditions that did not receive approved indication (Mackie et al., 2020; Olfson et al., 2015). There is also a concern for the disproportionate prescription of antipsychotic medications in youth from underserved communities (Mackie et al., 2020). Even when the prescription is justified, youth are thought to be physically and emotionally more vulnerable to antipsychotics' adverse effects because of their developing physiology and more exposed to stigma because of the negative impact on peer perceptions (Harrison et al., 2012). Evidence on safety outcomes is limited in children and adolescents and is often indirect or based on just one study (Krause et al., 2018). Moreover, little information does exist so far about the long‐term effects of antipsychotics on a still‐developing brain (Harrison et al., 2012). Nonetheless, amongst help‐seeking youth accessing early intervention services and deemed to be UHR for psychosis, the fraction, which has already been exposed to antipsychotic drug before enrollment is substantial: 20%–30% depending on the samples (Raballo et al., 2020a; Salazar de Pablo et al., 2020). According to some surveys, in general, dosages that are lower than the assumed minimum effective dose are used in these samples, and it has been suggested that in UHR patients antipsychotics are often used to treat comorbid disorders rather than emerging psychosis (Fusar‐Poli et al., 2020; Kotlicka‐Antczak et al., 2020). Indeed, UHR youth present substantial comorbidity with other mental disorders (Catalan et al., 2021). It should be noted; however, that current guidelines in the field discourage the use of antipsychotics as first‐line treatment in UHR patients, and, in particular, “any long‐term antipsychotic treatment with a primarily preventive purpose is not recommended” (Schmidt et al., 2015, p. 400).

Recent studies pointed to a negative prognostic impact of antipsychotics when prescribed to patients at high risk of psychosis, with an increased chance of conversion to psychosis after their prescription. In a sample of 83 participants diagnosed with a schizotypal disorder, Albert et al. (2017) found that treatment with antipsychotics at baseline was the most significant predictor of conversion to psychosis at a 3.5‐year follow‐up. In analysing data from the ShangHai At Risk for Psychosis (SHARP) study, Zhang, Xu, Tang, et al. (2020) found that UHR patients who did not receive antipsychotics showed a lower conversion rate than those who did (17.7% vs. 26.9%; odds ratio = 0.66; 95% confidence interval [CI] = 0.44, 0.98). Moreover, patients who initiated antipsychotic drugs whilst at clinical high risk of psychosis had lower remission rates than those who initiated antipsychotic drugs when already in the first episode of psychosis (Zhang, Xu, Wei, et al., 2020). In a meta‐analysis of 14 studies that reported detailed information on antipsychotics prescription in UHR patients, Raballo et al. (2020b) found that UHR patients who had received a prescription of antipsychotics before entering the programme of care (n = 112) had a higher relative risk (RR) of conversion to psychosis (29% vs. 16%; RR = 1.47; 95%CI: 1.18–1.83) than those who did not have received any (n = 235). Different interpretations were advanced to explain these findings. Albert et al. (2017) and Raballo et al. (2020b) suggested that treatment with antipsychotics is a proxy for elevated levels of psychiatric symptoms, thus marking a subgroup of individuals who have per se enhanced risk of conversion to psychosis. Zhang, Xu, Tang, et al. (2020); Zhang, Xu, Wei, et al. (2020), instead, suggested that antipsychotics should be considered harmful in subjects at high risk of psychosis, with no preventive benefits. For these reasons, Zhang, Xu, Tang, et al. (2020); Zhang, Xu, Wei, et al. (2020) discourage the prescription of antipsychotics in UHR patients unless presenting with a quite specific symptoms profile: severe positive and general symptoms, but mild negative symptoms.

So far, the evidence that the prescription of an antipsychotic drug is related to the conversion to psychosis in UHR people is limited. On one side, the start of the first treatment with antipsychotics is typically considered the endpoint of the DUP (Penttilä et al., 2014), thus it might be considered a functional equivalent of the conversion to psychosis in UHR patients (Raballo et al., 2020b; Raballo & Poletti, 2019). On the other side, in current clinical practise, antipsychotics are used beyond psychosis and, especially in developmental years they are prescribed off‐label to treat mood or anxiety symptoms as well as for the control of disruptive behavioural disorders (Olfson et al., 2015).

### 1.1 Aims

This study was set up to test whether the prescription of an antipsychotic drug during the treatment of help‐seeking people deemed to be UHR of psychosis is related to the subsequent risk of trespassing the psychometric threshold for psychosis. We expected that those who had received the prescription of antipsychotics during the first 2 years of treatment would be more likely to be found positive for psychosis according to a predefined psychometric threshold.

## 2 METHODS

Data were collected during the routine assessment of the patients participating in the Programma2000, the early intervention service operating under the Health Authority of the Niguarda Ca′ Granda Hospital of Milan (Cocchi et al., 2008). The study complies with the guidelines of the 1995 Declaration of Helsinki and its revisions (World Medical Association, 2013). Participants provided informed consent. The time interval of the study is from 1999 to 2015, when the Programma2000 was reorganized in both the assessment procedures and the therapeutic care pathways.

### 2.1 Participants

Referrals to Programma2000 arrive from institutionally mediated pathways (e.g., primary care, district Mental Health, school counselling, emergency rooms) but can be also self‐referrals from spontaneously help‐seeking individuals (Cocchi et al., 2013). The served catchment area includes approximately 200 000 inhabitants.

Criteria for referral and preliminary evaluation are: being aged up to 30 years old and help seeking for impending psychosis. Help‐seeking participants were initially screened with the Italian version of the Early Recognition Inventory Retrospective Assessment of Symptoms checklist (ERIraos‐CL). The ERIraos‐CL is a 17‐item screening checklist aimed at selecting persons in need of a more in‐depth assessment (Häfner et al., 1992; Raballo et al., 2014). Like the tool from which it derives, the ERIraos‐CL detects at‐risk mental states of psychosis with high sensitivity (Maurer et al., 2018; Rausch et al., 2013). Patients were deemed to be UHR for psychosis when they scored ≥12 on the ERIraos‐CL (Maurer et al., 2018) and met with the criteria of the Personal Assessment and Crisis Evaluation (PACE) Clinic in Melbourne for the identification of young people at incipient or “UHR” of developing a psychotic disorder (Yung et al., 2004; Yung & McGorry, 1996). Exclusion criteria were: previous antipsychotic treatment before referral; past or present diagnosis of psychosis in the spectrum of schizophrenia or in the affective spectrum (bipolar disorder, or unipolar disorder with psychotic features). A comorbid DSM‐IV or DSM‐IV‐TR diagnosis of substance dependence was an additional exclusion criterion, whilst substance use/abuse without dependence was not (Cocchi et al., 2008; Meneghelli et al., 2010).

### 2.2 Measures

The 24‐item Brief Psychiatric Rating Scale (BPRS) was used to quantify treatment response up to 2 years. The BRPS is a 24‐item measure of general psychopathology in a Likert format (from one [absent] to seven [extremely severe]), with higher total scores (ranging from 24 to 168) indicating higher levels of psychopathology (Overall & Gorham, 1962; Roncone et al., 1999). The BPRS was regularly administered to the patients at inception and then every 6 months, to assess levels of psychopathology and its change over time. Raters had a minimum of 2 years of experience in rating patients diagnosed with psychosis and an inter‐rater agreement, as measured as intra‐class correlation coefficients, of 0.70 or above when checked on a small sample of patients (n = 25). To measure conversion to psychosis in the sample, we applied the criteria for remission in schizophrenia (Andreasen et al., 2005). According to these criteria, item scores of mild or less (≤3 using the 1–7 range) on each of the target items of the BPRS for a 6‐month interval define the achievement of remission in patients with schizophrenia. We assumed, therefore, that scores higher than three on any of these target items would have been indicative of conversion to psychosis in UHR young people. The following seven target items were considered: grandiosity, suspiciousness, unusual thought content and hallucinatory behaviour, as indicative of psychoticism/reality distortion; conceptual disorganisation and mannerisms/posturing, as indicative of disorganisation; blunted affect, as indicative of negative symptoms and psychomotor poverty.

Participants were tested at a six‐month interval (i.e., every 26 weeks approximately) for 2 years. A participant was considered positive for conversion to psychosis when a score higher than three was marked on one or more of the seven BPRS target items as beforehand defined. It should be noted that the threshold for psychosis transition on the BPRS that was selected for this study is quite conservative and lower than the threshold habitually used on the Comprehensive Assessment of At‐Risk Mental States (CAARMS) or the Structured Interview for Prodromal Syndromes (SIPS). However, these tools were not available in Italy when Programma2000 was set up, and an Italian version was made available only later: 2011 for SIPS (Comparelli et al., 2011) and 2013 for CAARMS (Raballo et al., 2013). Nevertheless, the BPRS is an established measure of outcome in clinical trials, and it has been used to measure the effectiveness of psychosocial rehabilitation programmes (Inch et al., 1997) and to assess changes in psychopathology within UHR patients (Glenthøj et al., 2016).

Beside the BPRS, the following indicators were used in this study, as established on a detailed interview with the patient and a key informant, usually a close relative: gender (boy or girl); age at first contact (continuous, in years); duration of untreated illness (continuous, in months, defined as the interval between the onset of the first specific psychiatric symptom, whether or not related to psychosis, and the subsequent prescription of the first adequate pharmacological or psychological treatment); past admissions to hospital for psychiatric reasons (yes/no); substance use (yes/no); family history of psychiatric disorders (yes/no); premorbid functioning (yes/no, according to whether the patients have had a decline in their functioning at school/work or with their social relationship with peers in the past 2 years before contact with the centre); drop out of treatment after the first 2 years for any reason (yes/no).

We also checked the psychometric criterion for conversion to psychosis against the formal DSM‐IV or DSM‐IV‐TR diagnosis of schizophrenia‐spectrum psychosis made by the therapists at the end of the 3‐year programme. A psychiatrist made the diagnosis after a thorough revision of the clinical card and discussion with the team of treatment.

### 2.3 Treatment

During the study period treatment at the Programma2000 was based on a 3‐year comprehensive, tailored and flexible intervention package. The programme included individual psycho‐educational and motivational sessions, cognitive‐behavioural psychotherapy, individual family psycho‐education and support, therapeutic group activities (e.g., anxiety management, assertive and problem‐solving training, etc.), social group activities (e.g., music, multimedia, empowerment, computer training sessions, etc.), and supportive interventions on employment, school, compliance with medication, and planning of recreational activities (Cocchi et al., 2008; Meneghelli et al., 2010). Prescription of drugs was on an as‐needed basis, that is, when the treating staff decided that a patient might benefit from a drug, the drug was prescribed.

### 2.4 Statistics

Preliminary analysis was carried out using the Statistical Package for Social Sciences (SPSS) version 20. Additional analyses were carried out in R (R Core Team, 2018) using dedicated packages. All tests were two‐tailed, with alpha set at *p* < .05.

Two groups were compared: one group including all patients who received a prescription of antipsychotics at any time during the first 2 years of treatment (APs+), and a control group of patients who did not receive a prescription of antipsychotics during the treatment (APs‐). Initial comparisons were by chi‐square with Yates correction or Fisher exact test when n < 5 in any cell.

A non‐parametric Kaplan–Meier estimation with Cox proportional hazards model, both univariate and multivariate, including age (continuous) and sex (women as the referent group) as covariates, was used to calculate differences in survival between the APs + and APs − groups. Survival was calculated against the negative event of being positive for psychosis on target items of the BPRS as beforehand defined. Participants were censored if they did not convert to psychosis after 104 weeks (= 24 months, hence 2 years) of treatment. Violations of the proportionality assumption were assessed with the Schoenfeld Residuals Test. Survival analysis was carried out with the packages survival (Therneau, 2015) and survminer (Kassambara & Kosinski, 2018) running in R. The graphical representation of the results used the package ggplot2 (Wickham, 2016) running in R.

The accuracy of our BPRS threshold for predicting a formal DSM‐IV or DSM‐IV‐TR diagnosis of schizophrenia‐spectrum psychosis was assessed as the proportion of correct predictions (both true positives and true negatives) amongst the total number of cases examined and was expressed as area under the curve (AUC), which is a global measure of test performance, with 95% CI. Values of AUC between 0.80 and 0.90 are considered excellent, between 0.70 and 0.80 are considered acceptable (Altman et al., 2000).

## 3 RESULTS

The sample included 125 young people (aged between 16 and 30) who were identified as UHR for psychosis according to the predefined criteria. Table 1 summarizes the general characteristics of the sample.

**TABLE 1:** General characteristics of the sample (*n* = 125). All data are reported as mean (SD); range, or counts (percentage)

		Had reached the threshold for psychosis
Gender		
Boys	88 (70%)	23 (26%)
Girls	37 (30%)	8 (21%)
Age (years old)	22 (3); range: 16–30	
16–20 years old	53 (42%)	15 (28%)
21 years old or older	72 (58%)	16 (22%)
DUI (months)	30 (21); range: 1–60	
Less than 12 months	35 (51%)	9 (26%)
12 months or more	70 (35%)	18 (26%)
The DUI could not be determined	18 (14%)	
Past admissions for psychiatic reasons		
Yes	14 (11%)	5 (36%)
No	111 (89%)	26 (23%)
History of substance use		
Yes	22 (18%)	2 (9%)
No	85 (68%)	25 (29%)
Not enough information	18 (14%)	
Family history of psychiatric disorders		
Yes	67 (54%)	14 (21%)
No	40 (32%)	13 (32%)
Not enough information	18 (14%)	
Decline in premorbid functioning		
Yes	95 (74%)	20 (30%)
No	23 (18%)	7 (17%)
Not enough information	11 (8%)	
Dropout of treatment after 2 years		
Yes	38 (30%)	4 (10%)
No	87 (70%)	27 (31%)
BPRS		
Baseline	44 (11); range: 19–99	
At 6 months	37 (9); range: 24–76	24
At 12 months	33 (8); range: 24–76	4
At 18 months	33 (7); range: 24–62	2
At 24 months	32 (7); range: 24–59	1

Abbreviation: BPRS, Brief Psychiatric Rating Scale.

Overall, over the 2 years follow up, 30 (24%) subjects received the prescription of an antipsychotic (three subjects started with a typical antipsychotic then were switched to an atypical, second‐generation antipsychotic, like the others). Those who had received a prescription of antipsychotics during the study period did not differ in age, gender proportion or DUI from those who had not received one, but were more likely to have had a past admission to the hospital for psychiatric reasons (Table 2).

**TABLE 2:** Differences between participants who received a prescription of antipsychotics during the study period and those who did not. All data are reported as mean (SD); range, or counts (percentage)

	No antipsychotics	Antipsychotics	Statistics
	n = 95	n = 30	
Gender			
Boys	69 (73%)	19 (63%)	χ^2^_Yates_ = 0.55; df = 1; *p* = .46
Girls	26 (27%)	11 (37%)	
Age (years old)	22 (3); range: 16–30	21 (3); 17–30	t = 1.11; df = 52.5; *p* = .27^a^
16–20 years old	39 (41%)	14 (47%)	
21 years old or older	56 (59%)	16 (53%)	χ^2^_Yates_ = 0.11; df = 1; *p* = .74
DUI (months)	29 (21); range: 1–60	32 (22); 2–60	z = −0.41; *p* = .680^b^
Less than 12 months	25 (33%)	10 (33%)	χ^2^_Yates_ = 0.0; df = 1; *p* = 1.00
12 months or more	50 (67%)	20 (67%)	
Missing: n = 20			
Past admissions for psychiatic reasons			
Yes	7 (7%)	7 (23%)	χ^2^_Yates_ = 4.34; df = 1; *p* = .04
No	88 (93%)	23 (77%)	
History of substance use			
Yes	14 (18%)	8 (27%)	χ^2^_Yates_ = 0.50; df = 1; *p* = .48
No	63 (82%)	22 (73%)	
Missing: n = 18			
Family history of psychiatric disorders			
Yes	49 (64%)	18 (60%)	χ^2^_Yates_ = 0.01; df = 1; *p* = .90
No	28 (36%)	12 (40%)	
Missing: n = 18			
Decline in premorbid functioning			
Yes	45 (74%)	22 (30%)	χ^2^_Yates_ = 1.46; df = 1; *p* = .23
No	32 (18%)	8 (17%)	
Missing: n = 18			
Dropout of treatment			
after 2 years			
Yes	25 (26%)	13 (43%)	χ^2^_Yates_ = 2.37; df = 1; *p* = .12
No	70 (74%)	17 (57%)	
BPRS			
Baseline	43 (12); range: 19–99	47 (10); range: 29–68	z = −2.32; *p* = .020^b^
At 6 months	36 (10); range: 24–76	40 (7); range: 29–55	z = −2.95; *p* = .003^b^
At 12 months	32 (8); range: 24–76	34 (6); range: 24–54	z = −1.24; *p* = .214^b^
At 18 months	31 (7); range: 24–62	33 (7); range: 24–56	z = −2.12; *p* = .034^b^
At 24 months	31 (6); range: 24–59	31 (5); range: 24–41	z = −0.92; *p* = .356^b^

a Welch's t‐test (because of unequal variance).

b Non‐parametric Mann–Whitney U test.

The main difference between the two groups concerned the scores on the BPRS. Those who had received antipsychotics had higher scores on the BPRS at baseline, at 6 months, and at 18 months, suggesting that they were modestly more severe at inception and showed greater levels of psychopathology also during the study period.

At the end of the first 2‐year period of treatment, in the sample there were 31 participants (25%) who had reached the psychometric threshold for conversion to psychosis. Those who had received a prescription of antipsychotics during the study period were more likely to have reached the threshold for conversion to psychosis (14 out of 30 [47%]) than those who did not receive a prescription of antipsychotics (17 out of 95 [18%]): χ^2^_Yates_ = 8.63; df = 1; *p* = .003.

In the sample eight UHR patients received a formal diagnosis of schizophrenia‐spectrum psychosis at the end of the three‐year programme, five (out of 31; 16%) amongst those who reached the psychometric threshold for conversion to psychosis at the end of the first 2 years of treatment and three (out of 94; 3%) amongst those who did not (Fisher exact test: *p* = .022). Predictive accuracy of the BPRS threshold was 0.76 (95% CI: 0.68–0.84).

### 3.1 Survival analysis

**FIGURE 1:**
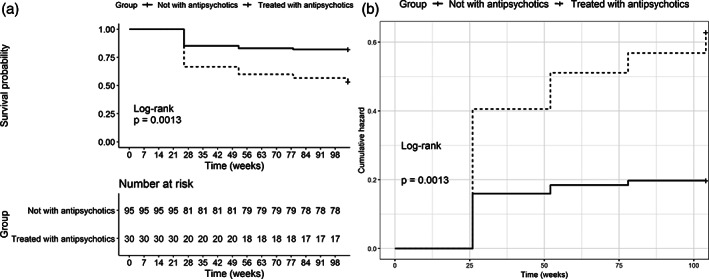
Survival curves with the number at risk by time, based on (a) Kaplan–Meier estimates of the negative event as representative of positivity for psychosis on the BPRS according to the prescription of antipsychotics (+ or –) and (b) cumulative hazard of the positivity for psychosis on the BPRS of the same data. BPRS, Brief Psychiatric Rating Scale

UHR people who received a prescription of antipsychotics during the first 2 years of treatment were statistically more likely to reach the psychometric threshold for conversion to psychosis on the BPRS at follow‐up: Hazard ratio (HR) = 3.03 (95%CI: 1.49–6.16); Likelihood ratio test: 8.69; d.f. = 1; *p* = .003; log‐rank test: 10.43; *p* = .001 (Figure 1).

No evidence against the proportionality assumption could be found (Schoenfeld Residuals Test: χ^2^ = 0.25, *p* = .61). The Cox regression analysis confirmed a greater chance of reaching the psychometric threshold for conversion to psychosis on the BPRS amongst those who received a prescription of antipsychotics than amongst those who did not receive it, even when sex and age were accounted for (Table 3).

**TABLE 3:** Univariate and multivariate cox proportional regression analyses of the primary endpoint (psychometric conversion to psychosis) in the sample (n = 125)

	HR	95%ci	*p*‐value	Likelihood ratio	Schoenfeld residuals test
Univariate					
AP+	3.03	1.49–6.16	.002	LR = 8.69; *p* = 0.003	χ^2^ = 0.25, *p* = .61
Multivariate First analysis					
AP+	3.11	1.52–6.37	.002	LR = 9.46; *p* = 0.02	χ^2^ = 0.98, *p* = .322
Sex	1.30	0.61–3.11	.437		χ^2^ = 0.86, *p* = .353
Age	0.98	0.88–1.09	.771		χ^2^ = 2.32, *p* = .127
Multivariate Second analysis					
AP+	3.92	1.73–8.91	.001	LR = 24.59; *p* = 0.003	χ^2^ = 0.31, *p* = .578
Sex (boys)	1.24	0.50–3.07	.640		χ^2^ = 0.34, *p* = .561
Age (continuous)	0.98	0.87–1.09	.692		χ^2^ = 1.54, *p* = .214
DUI (continuous)	0.99	0.97–1.01	.576		χ^2^ = 0.01, *p* = .925
Past admissions for psychiatic reasons (yes)	2.45	0.67–8.92	.173		χ^2^ = 0.06, *p* = .799
History of substance use (yes)	0.23	0.04–1.11	.068		χ^2^ = 0.09, *p* = .757
Family history of psychiatric disorders (yes)	0.52	0.22–1.23	.136		χ^2^ = 1.40, *p* = .236
Dropout of treatment after 2 years (yes)	0.32	0.10–0.99	.049		χ^2^ = 1.34, *p* = .247
Decline in premorbid functioning (yes)	1.60	0.58–4.42	.360		χ^2^ = 0.01, *p* = .971

*Note:* AP+ = Had received the prescription of an antipsychotic during the first 2 year of treatment.

When taking into account factors that might impact on the primary outcome, participants who drop out of treatment after the first 2 years (HR = 0.32; 0.10–0.99, *p* = .049) and those with a history of substance use (HR = 0.23; 0.04–1.11, *p* = .068) were marginally less likely to reach the psychometric threshold for conversion to psychosis on the BPRS. Again, no violation against the proportionality assumption could be found (see Table 3).

## 4 DISCUSSION

This study showed that a prescription of antipsychotics in UHR is statistically linked to a greater chance of reaching a psychometric threshold for conversion to psychosis at follow‐up. The most obvious explanation is that UHR people who show impending signs of more severe psychopathology are more likely to receive a prescription of antipsychotics by the treating clinicians who aim at decreasing symptoms' impact and avert the risk of conversion to psychosis. In the sample, those who had received antipsychotics had modestly higher scores on the BPRS at baseline and 6 months and again at 18 months. This might suggest that they were more likely to show symptoms indicative of possible conversion to psychosis or anyhow a slightly more severe overall psychopathological outlook although still below the formal psychometric threshold for conversion to psychosis. Indeed, the naturalistic threshold adopted in this study is the one at which the prescription of antipsychotics is expected to be started in clinical practise (presence of persisting and non‐negligible symptoms of psychosis). The finding suggests that the prescription of an antipsychotic is indeed a marker of possible conversion to psychosis in UHR patients, and that within UHR populations the intercurrent prescription of antipsychotics could be considered a functional equivalent of such conversion (Raballo et al., 2020b; Raballo & Poletti, 2019).

This result is corroborated by the observation that those who dropped out of treatment before the termination of the three‐year programme were less likely to reach the psychometric threshold for conversion to psychosis. This may depend on people dropping out of treatment when they are not so severe to instigate scrutiny and assertive outreach after their anticipated conclusion of the programme. Essentially, people with more severe symptoms are less likely to leave the treatment since they receive more intensive care according to the tailored protocol. Also those with a history of substance use were marginally less likely to reach the psychometric threshold for conversion to psychosis. These patients may have benefitted from a combination of the tailored protocol of care for the UHR symptoms with a dedicated treatment for substance use/abuse. The closer scrutiny resulting from the greater attention to the risk of psychosis in those with substance use (Rapado‐Castro et al., 2015) may have prevented the emergence of severe symptoms of psychosis in this specific subgroup. For example, UHR patients often have a history of cannabis use (Farris et al., 2020), and those with a history of cannabis use have more severe symptoms of psychosis (Carney et al., 2017). However, despite being frequently related to conversion to psychosis, cannabis use was not statistically associated with conversion to psychosis in a recent meta‐analysis: RR = 1.11, 95% CI = 0.89–1.37 (Farris et al., 2020). The reason may be that substance use, being a well‐known marker of risk for psychosis (Rapado‐Castro et al., 2015), prompts the application of more intensive care that may eventually protect against the risk of conversion to psychosis.

In this study, we have used a threshold for conversion to psychosis based on the BPRS that is lower than the threshold habitually used on the CAARMS or the SIPS. Nevertheless, the predictive accuracy of our procedure, when tested against a formal clinical diagnosis of schizophrenia‐spectrum psychosis, resulted comparable to the estimated accuracy of the CAARMS (AUC = 0.79 95% CI: 0.75–0.83; Oliver et al., 2018) and marginally lower than the one of the SIPS (AUC = 0.80; 95% CI: 0.66–0.95; Zhang et al., 2019).

### 4.1 Strengths and limitations

The main strength of the study is the availability of longitudinal data that was prospectively collected for purposes unrelated to the aims of the study thus limiting the risk of bias in the subsequent analysis. Although BPRS is a suboptimal tool for the clinical profiling of UHR help‐seekers, the psychometric threshold that we used to define the conversion to psychosis showed an acceptable relationship with the formal diagnosis for schizophrenia‐spectrum psychosis as formulated by the treating staff at the end of the three‐year programme of care, thus corroborating its validity. However, we were unable to have temporal data about the prescription of the antipsychotics. We only had the information about whether or not a patient received the prescription of an antipsychotic during the first 2 years of treatment, thus we were unable to precisely relate the prescription of the antipsychotic to the later trespassing of the psychometric threshold for conversion to psychosis.

## 5 CONCLUSIONS

The intercurrent prescription of antipsychotics in UHR individuals attending Programma 2000 was statistically linked to a higher risk of trespassing the psychometric threshold for conversion to psychosis. When psychopathology becomes more severe and subthreshold symptoms of psychosis more conspicuous, treating clinicians tend to prescribe antipsychotics to mitigate psychopathological worsening. Thus, antipsychotic prescription in UHR patients would be a proxy for a higher severity subgroup with stronger potential to develop psychosis. However, we cannot exclude that for some patients, the prescription of an antipsychotic drug might prompt a sensibilization of the dopamine receptors, which may favour a further rebound of symptoms until conversion to psychosis (Chouinard et al., 2017). In‐depth scrutiny of the symptoms' profile is thus necessary to wisely ponder the best, individualized treatment option for UHR patients (Zhang, Xu, Tang, et al., 2020; Zhang, Xu, Wei, et al., 2020), balancing psychopharmacotherapy with psychotherapy approaches, such as cognitive‐behavioural therapy, which offers first‐line protection from conversion to psychosis (Devoe et al., 2020).

## CONFLICT OF INTEREST

The authors declare no conflicts of interest.

## FINANCIAL SUPPORT

None for this study.

## Data Availability

Research data are not shared since informed consent only allowed analysis and publication of collected data as a summary or group description.
